# A New Fragment‐Based Pharmacophore Virtual Screening Workflow Identifies Potent Inhibitors of SARS‐CoV‐2 NSP13 Helicase

**DOI:** 10.1002/jcc.70201

**Published:** 2025-09-05

**Authors:** Jordi Doijen, Jiexiong Xie, Simone Marsili, Trpta Bains, Mandeep Kaur Mann, Pravien Abeywickrema, Nick Van den Broeck, Christian Permann, Thierry Langer, Gökhan Ibis, Charles‐Alexandre Mattelaer, Jeremy Harvey, Sebastiaan van Raalte, Roberto Fino, Vineet Pande, Danielle Peeters, Aaron Patrick, Ellen Van Damme, Herman van Vlijmen, Marnix Van Loock, Edgar Jacoby

**Affiliations:** ^1^ Johnson & Johnson Beerse Belgium; ^2^ Johnson & Johnson Toledo Spain; ^3^ Johnson & Johnson Spring House Pennsylvania USA; ^4^ Charles River Laboratories Beerse Belgium; ^5^ Department of Pharmaceutical Sciences University of Vienna Vienna Austria; ^6^ Inte:Ligand Software‐Entwicklungs Und Consulting GmbH Vienna Austria; ^7^ Department of Chemistry KU Leuven Leuven Belgium

**Keywords:** broad‐spectrum antiviral, fragment‐based, FragmentScout, SARS‐CoV‐2 NSP13 helicase, virtual screening

## Abstract

Herein we report the in silico discovery of 13 novel micromolar potent inhibitors of the SARS‐CoV‐2 NSP13 helicase validated in cellular antiviral and biophysical ThermoFluor assays. The compounds, discovered using a novel fragment‐based pharmacophore virtual screening workflow named FragmentScout, enable the advancement of novel antiviral agents. FragmentScout uses publicly accessible structural data of the SARS‐CoV‐2 NSP13 helicase, which was previously generated at the Diamond LightSource by XChem high‐throughput crystallographic fragment screening. The workflow generates a joint pharmacophore query for each binding site, thereby aggregating the pharmacophore feature information present in each experimental fragment pose. The joint pharmacophore query is then used to search 3D conformational databases using the Inte:ligand LigandScout XT software. The FragmentScout in silico workflow offers a novel tool for identifying micromolar hits from millimolar fragments in fragment‐based lead discovery. It is anticipated that this workflow will enhance systematic data mining of the growing collection of XChem datasets.

## Graphical abstract

The new fragment‐based pharmacophore virtual screening workflow FragmentScout is introduced, which aggregates feature information present in each experimental fragment pose from XChem high‐throughput crystallographic screening. The method is applied to the SARS‐CoV‐2 NSP13 helicase, and we report the in silico discovery of 13 novel micromolar potent inhibitors with broad‐spectrum single‐digit micromolar activity in cellular antiviral assays.

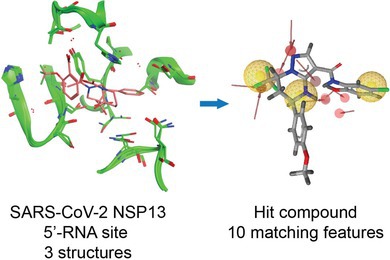

## 1 Introduction

XChem primary screening, a high‐throughput crystallographic screening of fragments technology, was introduced in 2014 by the von Delft group to accelerate the fragment‐based lead‐discovery process [1]. This workflow has been applied to over a 100 targets from diverse therapeutic areas. This method effectively identified weak binders which both serve as high‐quality starting points for compound design and provide extensive structural information on binding sites. The XChem facility at the Diamond Light Source, for large‐scale crystallographic fragment screening, is accessible to academic and industrial users worldwide. It has especially been impactful in drug discovery against SARS‐CoV‐2 [1, 2, 3]. A key bottleneck in fragment‐based lead discovery is the evolution of a primary fragment hit with millimolar potency to a lead candidate with micromolar potency in a biophysical assay.

The development of the structural cheminformatics platform Fragalysis [4] by the same group enables the analysis of the data and the follow‐up of individual fragments. Searching for chemical structures of expanded analogues for each fragment in ultra‐large chemical databases, such as the Enamine REAL database, is facilitated by newly introduced graph‐based chemical database technology [5]. As XChem typically identifies a variety of clusters of multiple fragments with affinity to distinct binding sites on the target, we envisioned there would be a complementary opportunity in developing and applying a new pharmacophore searching workflow called FragmentScout for each such cluster. Using Inte:ligand's LigandScout software [6], we combined the pharmacophore feature information present in every experimental fragment pose and aggregate this information in one joint pharmacophore query for each binding site cluster. The joint pharmacophore query is then used to search large 3D conformational databases. This publication introduces the methodology and applies it prospectively to the SARS‐CoV‐2 NSP13 helicase, for which recently released XChem Data has been generated [7]. SARS‐CoV‐2 is the causative agent of the 2019–2020 COVID‐19 pandemic [8]. The SARS‐CoV‐2 helicase NSP13 is a member of the helicase super‐family 1B and a key component of the SARS‐CoV‐2 replication machinery [9, 10, 11]. By ATP hydrolysis, NSP13 catalyzes the unwinding of double‐stranded DNA or RNA in a 5′–3′ direction. NSP13 is part of the replication‐transcription complex by interacting with the viral RNA‐dependent RNA polymerase NSP12. In addition to its helicase activity, NSP13 also possesses RNA 5′ triphosphatase activity, suggesting a further role for NSP13 in the formation of the viral 5′ mRNA cap [7, 12]. Due to its essential role in viral replication and because of the high conservation of NSP13 across coronavirus species, the helicase has been suggested as a promising target for the development of new antiviral drugs [13, 14]. Recently, NSP13 was the target of various virtual screening approaches [15, 16, 17, 18, 19] and was selected as the Challenge #2 target for the CACHE competition where participants are asked to find hits for the NSP13 RNA‐binding site using diverse virtual screening approaches [20].

The performance of our new FragmentScout fragment‐based pharmacophore virtual screening workflow was compared with more classical docking‐based virtual screening using the Glide docking software [21, 22]. Through the FragmentScout workflow, we describe the discovery of novel micromolar potent SARS‐CoV‐2 NSP13 helicase inhibitors, both validated in cellular antiviral and biophysical ThermoFluor assays, opening the way to further development of novel antiviral agents.

## 2 Methods

### 2.1 The SARS‐CoV‐2 NSP13 Helicase Dataset

Fifty‐one XChem PanDDA NSP13 fragment screening crystallographic coordinate files [7] deposited in the RCSB protein data bank [23, 24] with the following accession codes were downloaded and included in this analysis:

5RL6, 5RL7, 5RL8, 5RL9, 5RLB, 5RLC, 5RLD, 5RLE, 5RLF, 5RLG, 5RLH, 5RLI, 5RLJ, 5RLK, 5RLL, 5RLM, 5RLN, 5RLO, 5RLP, 5RLQ, 5RLR, 5RLS, 5RLT, 5RLU, 5RLV, 5RLW, 5RLY, 5RLZ, 5RM0, 5RM1, 5RM2, 5RM3, 5RM4, 5RM5, 5RM6, 5RM7, 5RM8, 5RM9, 5RMA, 5RMB, 5RMC, 5RMD, 5RME, 5RMF, 5RMG, 5RMH, 5RMI, 5RMJ, 5RMK, 5RML, 5RMM.

In addition, the 6XEZ cryo‐EM structure of the SARS‐CoV‐2 replication‐transcription complex was included [9]. The coordinates of the E chain NSP13 molecule were extracted, including one copy of a bound ATP‐mimetic ligand.

### 2.2 Detection of Pharmacophore Features and Generation of a Joint Pharmacophore Query per Binding Site

The generation of the joint pharmacophore query was set up in an interactive mode using the LigandScout 4.5 software. The process started with a set of 3D structurally pre‐aligned protein data bank (PDB) files. Each structure was imported into the structure‐based perspective of the software. The pharmacophore feature assignment, the addition of the exclusion volumes, and the addition of the exclusion volumes coat (i.e., a second shell of exclusion volumes) were done automatically. The generated pharmacophore query was subsequently stored in the alignment perspective of the software. This process was repeated for all structures of a given binding site. Within the alignment perspective, all queries were selected, aligned, and merged using the based‐on reference points option. A final step consisted of the interpolation of all features within a distance tolerance, which resulted in the joint pharmacophore query for this binding site.

### 2.3 LigandScout XT Pharmacophore‐Based Virtual Screening

In LigandScout XT virtual screening of chemical compound libraries using chemical feature‐based pharmacophore models as search queries, fitting molecules are identified by a new alignment algorithm, without the need for a pre‐filtering step [6, 25]. This makes the procedure useful for ultra‐large libraries, where file space becomes an issue. The Greedy 3‐Point Search aims at finding optimal alignments by using a matching‐feature‐pair maximizing search strategy, thus being faster and more accurate than previously existing methods [26]. This opens the possibility for fragment screening with a minimum number of required features, which is drastically smaller than the total number of features in LigandScout XT, as this was previously not computationally feasible. This is especially relevant for the models from the FragmentScout workflow, as the combined fragment models result in quite large models (e.g., 22 features). The joint pharmacophore query generated, as described above, was used for virtual screening of the J&J internal screening compound collection, which had been converted into the LigandScout ldb2 format using the CONFORGE conformer generator [27].

### 2.4 Glide Docking

Glide docking approximates a complete systematic search of the conformational, orientational, and positional space of the docked ligand in a rigid protein receptor binding pocket [21, 22]. An initial rough positioning and scoring phase that dramatically narrows the search space is followed by torsionally flexible energy optimization on an OPLS3‐based non‐bonded potential grid for a few 100 surviving candidate poses. The very best candidate poses are further refined via Monte Carlo sampling. The selection of the best docking pose for each compound is done using the model energy function GlideScore that combines empirical and force‐field‐based terms.

Two high‐resolution NSP13 protein structures were used for docking in the NSP13 nucleotide (PDB: 5RL7, 1.89 Å resolution) and the 5′‐RNA (PDB: 5RLZ, 1.97 Å resolution) pockets. Protein and ligands were, respectively, prepared with the Protein Preparation Wizard and LigPrep using default settings. Water molecules within the 5 Å contact sphere of the ligand were retained. For the docking parameters, Glide was run in Standard Precision (SP) mode. For the nucleotide binding pocket, five hydrogen bond constraints were defined corresponding to hydrogen bonds with donors from the side chains of Arg442, Arg443, and Lys320, and the backbone NH groups of Gly287 and Lys288. Similarly, three hydrogen bond constraints were defined for the 5′‐RNA pocket, corresponding to donors from the side chains of Ser486 and Asn516, and the backbone NH group of Asn516. For both pockets, the generated docking poses had a docking score lower than −7 kcal/mol and formed at least one of the interactions defined in the corresponding list. Finally, selected Glide hits for both the nucleotide and the 5′‐RNA pockets have docking scores < −8 kcal/mol, respectively, and ligand_efficiency_ln < −1.8 and −1.9 kcal/mol respectively. Ligand_efficiency_ln is calculated as shown in the following equation:

(1) $$\;\text{Ligand}\_\text{efficiency}\_\ln=\frac{\text{Docking score}}{\left(1+\ln\left(\mathrm{no}.\;\text{of heavy atoms}\right)\right)}$$

The cutoffs were chosen as the average ligand_efficiency_ln value for docking poses with a docking score of −8 kcal/mol for the nucleotide and 5′‐RNA pockets.

### 2.5 In Vitro Assays

#### 2.5.1 Protein Production and Purification

SARS‐CoV‐2 NSP13 (GenBank: MN908947) gene was cloned into a pvL1393 vector with an added N‐terminus His6 tag, SUMOStar tag, and HRV3C protease cleavage site (MG‐HHHHHH‐GSLQDSEVNQEAKPEVKPEVKPETHINLKVSDGSSEIFFKIKKTTPLRRLMEAFAKRQGKEMDSLTFLYDGIEIQADQTPEDLDMEDNDIIEAHREQIGG‐LEVLFQGP). NSP13 was expressed in Tni hi5 cells and collected 48 h post‐infection by centrifugation. Cell pellets were re‐suspended in Buffer 1 (50 mM Tris pH 8.0, 500 mM NaCl, 10% glycerol (v/v), 1 mM TCEP, Complete protease inhibitor cocktail) and homogenized by sonication. Supernatant was collected by centrifugation and incubated with pre‐equilibrated cobalt resin at 4°C for 1 h. The resin was washed with 10 CV Buffer 2 (50 mM Tris pH 8.0, 1 M NaCl, 10% glycerol (v/v), 1 mM TCEP) and 10 CV of Buffer 3 (50 mM Tris pH 8.0, 300 mM NaCl, 10% glycerol (v/v), 1 mM TCEP). The protein was eluted with Buffer 4 (50 mM Tris pH 8.0, 300 mM NaCl, 10% glycerol (v/v), 1 mM TCEP, 20/50/250 mM imidazole). HRV3C protease was added to the eluate with a weight ratio of 1:100 (enzyme: substrate) and dialyzed overnight at 4°C in Buffer 5 (50 mM Tris pH 8.0, 300 mM NaCl, 10% glycerol (v/v), 1 mM TCEP). The digested protein was incubated with cobalt resin at 4°C for 30 min, and the flow‐through was collected and further purified on a Superdex 200 10/300 column in Buffer 6 (20 mM Tris pH 8.0, 300 mM NaCl, 10% glycerol (v/v), 1 mM TCEP). Purified NSP13 was concentrated to 5 mg/mL and stored at −80°C.

#### 2.5.2 Nsp13 ThermoFluor Assay

ThermoFluor is a fluorescence‐based biophysical technique that can be used to assess protein folding states and stability over gradual, continuous increases in temperature. As the protein starts to unfold over a temperature range, a dye that is sensitive to an aqueous environment binds to the exposed hydrophobic regions and fluoresces [28]. Melting temperature (*T*_m_) values are obtained at the midpoint of an unfolding transition, and the addition of ligands can lead to further stabilization. Shifts in melting points described as Δ*T*_m_ are proportional to the affinity of the ligand tested [29].

SARS‐CoV‐2 NSP13 was diluted to a 0.1 mg/mL protein concentration in buffer containing 5X SYPRO Orange dye (Sigma), 1X HBSN (Cytiva), 3 mM MgCl2 (Sigma), 0.5 mM TCEP (Thermo Fisher), at pH 7.4. A volume of 4 μL/well of protein/dye was dispensed into assay plates containing compounds to be screened at a final assay concentration of 33 μM. Protein melting curves were obtained using a Light Cycler 480 II (Roche) instrument across a 25°C–95°C temperature gradient. Compounds with a Δ*T*_m_ ≥ 0.6°C are considered to show statistically significant stabilization as this corresponds to a Δ*T*_m_ result equal to or greater than three times the standard deviation of Apo NSP13, which was found to be 0.2°C across 430 sample wells. These compounds were then followed up in dose response starting at 400 or 200 μM with twofold dilutions across seven points.

#### 2.5.3 Cell Lines and Viruses

A549‐hACE2 cells (InvivoGen) were cultured in RPMI‐1640 (Gibco) supplemented with 10% heat‐inactivated fetal bovine serum (FBS; Biowest), 2 mM alanyl‐glutamine (Sigma), 20 μg/mL gentamicin (Gibco) and 0.5 μg/mL puromycin (Gibco). HeLa‐hACE2 cells (Creative Biogene) were grown in Dulbecco's Modified Eagle's Medium (DMEM; Gibco) supplemented with 10% heat‐inactivated FBS, 2 mM alanyl‐glutamine, 20 μg/mL gentamicin, and 0.5 μg/mL puromycin. Puromycin was omitted from the assay media. Cells were maintained at 37°C in 5% CO_2_.

A Belgian strain of SARS‐CoV‐2 (SARS‐CoV‐2 B1 [BetaCov/Belgium/GHB‐03021/2020 lineage A, B.1]) [30] was obtained from the Rega Institute for Medical Research (KU Leuven), Belgium. SARS‐CoV (BetaCoV/SARS‐CoV‐1/FFM1) was obtained from Goethe University Frankfurt, Germany. HCoV‐229E (AlphaCoV/VR‐740) was obtained from ATCC. Viral stocks were generated and titrated in Vero E6 cells [30], except for HCoV‐229E (Huh7 cells). Viral stocks were frozen at −80°C.

#### 2.5.4 Library

A proprietary compound library at Janssen Pharmaceutica NV consisting of ~1.5 M small‐molecule compounds was used for virtual screening.

Compounds selected through the virtual screening effort are named virtual screening (VS) hits. When the activity of a VS hit is confirmed at a single dose in the cellular assay or at a single dose in the ThermoFluor assay, it is seen as a primary hit. When activity is confirmed in a dose response according to the preset selection criteria in both the SARS‐CoV‐2 cell‐based and NSP13 ThermoFluor assays, the compound is a validated hit.

#### 2.5.5 Reference Compounds

Remdesivir and PF‐07321332 (Nirmatrelvir) were used in the cellular confirmation assays as reference compounds with known antiviral activity against SARS‐CoV‐2. For remdesivir, the prodrug GS‐5734 with CAS 1809249‐37‐3 was used in all experiments. Remdesivir and Nirmatrelvir were included as they are well‐known and clinically relevant antivirals for SARS‐CoV‐2 that respectively act on the well‐characterized targets RdRp and Mpro [31, 32, 33]. Ponatinib was used as a positive control for toxicity testing in A549‐hACE2.

#### 2.5.6 Viral Infection

SARS‐CoV‐2 B1 and SARS‐CoV were used to infect A549‐hACE2 cells, whereas HCoV‐229E was used to infect HeLa‐hACE2 cells, as this HCoV uses human aminopeptidase N (hAPN) as a receptor [34] which is expressed on HeLa‐hACE2 cells but not on A549‐hACE2. Experiment media followed the same composition as for cell cultivation but omitted selection antibiotics. 384‐well assay plates (Phenoplate, Revvity) were pre‐spotted with dimethyl sulfoxide (DMSO)‐dissolved compound using the Echo Acoustic liquid handler (Labcyte). For SARS‐CoV‐2 B1 and SARS‐CoV antiviral testing, A549‐hACE2 cells were seeded in assay medium at 7000 cells/well and immediately inoculated at an MOI of 0.175 or 0.02, respectively. The assay was incubated for 24 h at 37°C in a humidified CO_2_ incubator.

For the hCoV‐229E assay, HeLa‐hACE2 cells were seeded at a density of 4000 cells/well and directly challenged with virus at MOI 0.8. Plates were incubated for 48 h at 35°C, 5% CO_2_.

#### 2.5.7 High‐Content Imaging (HCI) Antiviral Assays

Fixation, staining, and HCI followed the methods described in [35].

In brief, infected cultures were fixated using methanol‐free formaldehyde (Polyscience) at a final concentration of 3–4 v/v% and incubated for 15 min at room temperature. Following fixation, plates were washed and treated for 2 h with a cocktail of permeabilizing and blocking agents, and primary monoclonal antibodies targeting SARS‐CoV(−2) spike protein (rabbit IgG) or double‐stranded RNA (dsRNA, mouse IgG2a) as a generic marker for hCoV‐229E infection, respectively. As a second staining step, plates were treated with a mixture of stains for downstream nuclei and cell body segmentation (Hoechst 33,342 and HCS Cell Mask Deep Red, Invitrogen) and secondary goat‐derived polyclonal antibodies conjugated to Alexa Fluor 568 or Alexa Fluor 488 (Invitrogen) for visualization of sarbecovirus spike protein and hCoV‐229E dsRNA structures, respectively. Plates were imaged using a Cell Voyager 8000 confocal microscope (Yokogawa) using pre‐defined acquisition parameters. The collected image data was transferred to Phaedra HCI analysis software and processed further by means of calculating the percentageofinfection using the mean signal intensities obtained for infected and non‐infected DMSO controls, respectively. In turn, these percentages were used to calculate 50% effective concentrations (EC_50_) and to construct dose–response curves which were analyzed further in Graphpad Prism (version 10).

For the single dose cell‐based SARS‐CoV‐2 assay, ≥ 30% inhibition in spike at 10 μM of compound was set as the criterion for hit selection to ensure that it would be possible to obtain an EC_50_ value in the dose response follow‐up. Thirty percent inhibition of spike positive cells is considered significant as inhibition with 6% or more corresponds to three times the standard deviation of the infected control (0% inhibition) measured across 383 wells. Selected compounds were followed up in dose response starting at 25 or 12.5 μM with three‐fold dilutions across 11 points.

#### 2.5.8 Toxicity Assay

A549‐hACE2 cells were seeded in compound pre‐spotted plates at a density of 4000 cells/well in 384‐well plates (Phenoplate, Revvity). After 24 h incubation of the plates, 1‐step ATPlite reagent (Perkin Elmer) was prepared according to the manufacturer's instructions and added in a 1:1 ratio, followed by an incubation in the dark on an orbital shaker at 700 rpm for 2 min at room temperature, followed by a short spin. ATP‐generated chemiluminescence was measured using the ViewLux plate reader (Perkin Elmer).

## 3 Results and Discussion

### 3.1 Outline of the FragmentScout—Fragment‐Based Pharmacophore Virtual Screening Workflow

**FIGURE 1:**
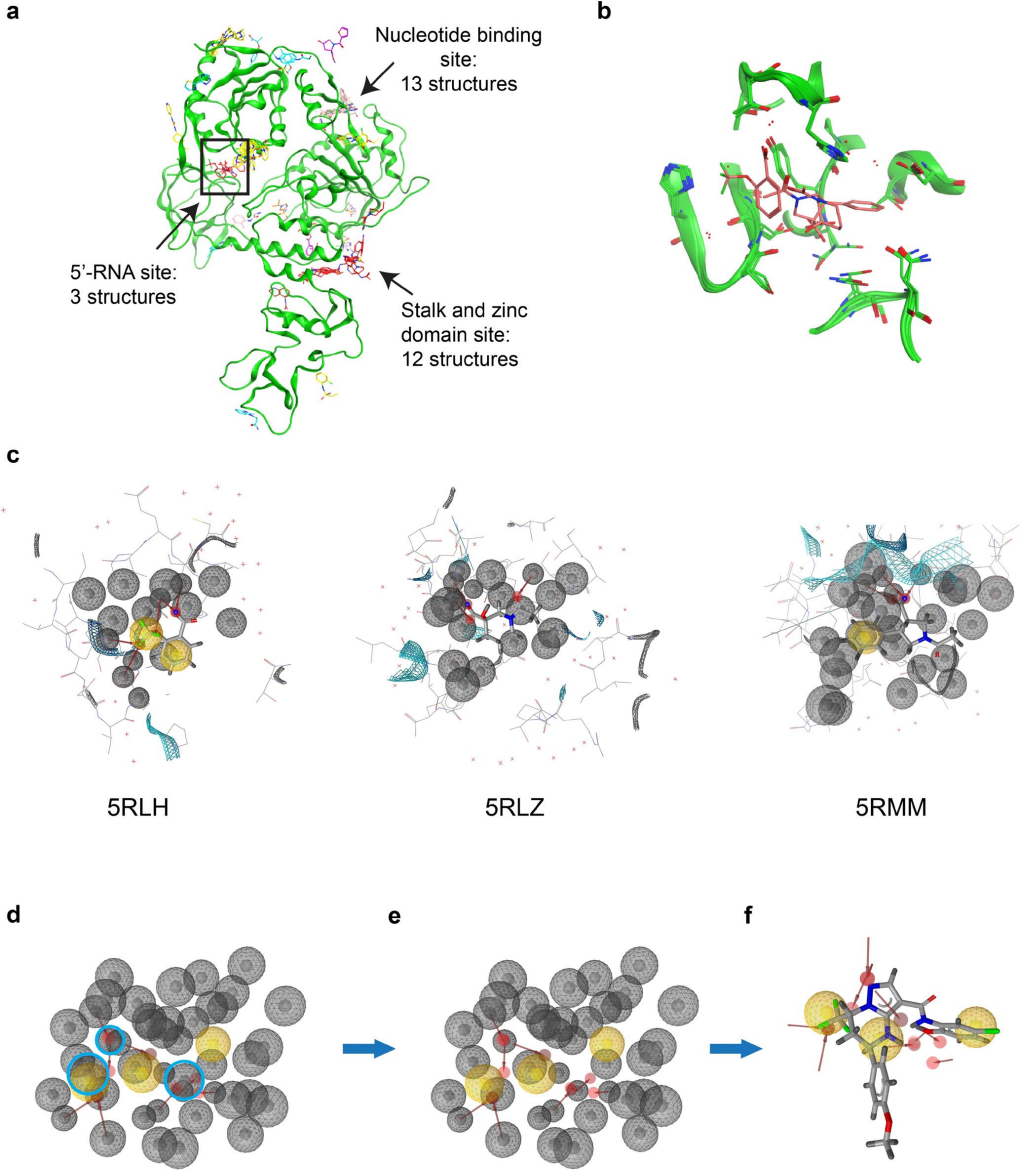
Outline of the FragmentScout—fragment‐based pharmacophore virtual screening workflow. (a) The 5RL6 NSP13 structure with superimposition of the 63 retained fragment poses of the XChem dataset [7] which cluster in 19 different binding sites of which three are highlighted: The nucleotide binding site, the 5′‐RNA binding site (black box), and the stalk‐zinc interdomain binding site. (b) The superimposition of the 5RLH, 5RLZ and 5RMM structures with fragments binding in the 5′‐RNA site. (c) The pharmacophore queries for the individual 5RLH, 5RLZ, and 5RMM structures. (d) and (e) The joint pharmacophore query for the 5′‐RNA binding site. Highlighted are hydrophobic (yellow) and H‐Bond acceptor (red) pharmacophore features and the protein excluded volume features (gray). The excluded volume features highlighted by the cyan circles in (d) were removed in (e) and are occluding the steric access to other pharmacophore features of the joint query. (f) An example of a hit compound matching 10 of the 12 pharmacophore query features.

The FragmentScout workflow consists of three sequential steps: first, it involves conducting supervised cluster analysis of the XChem dataset; next, it entails selecting binding sites for pharmacophore analysis and generating a combined pharmacophore query for each binding site; finally, it includes performing virtual screening with LigandScout pharmacophores. The workflow is illustrated in Figure 1 for the SARS‐CoV‐2‐NSP13 helicase.

For our new internal workflow, a total of 51 fragment‐protein complexes were prepared using CCG MOE2022.2 software. Given that 9 PDB files contain two or three copies of a given fragment in the asymmetric unit containing itself two NSP13 molecules, the coordinate files were split to generate 63 NSP13 monomer structures binding each one single fragment. The 63 fragment‐monomer pair structures were then 3D‐superimposed in the CCG MOE2022.2 software using the 5RL6 structure as a reference. Supervised visual clustering was then performed using the same software by importing the fragment‐monomer pairs one‐by‐one in alphanumeric order and attributing, depending on the spatial overlap, a new or already existing binding site number to each newly imported fragment‐monomer complex. Clusters that started to overlap spatially were merged. This process, which can potentially be further automated, yielded 19 binding site clusters each counting between 1 and 13 fragments.

Three of the 19 binding sites were selected for further analysis. This selection includes two pockets previously identified by Newman et al. that are expected to be functionally relevant, namely the nucleotide binding site located between the 1A and 2A domains (13 fragment structures plus ATP mimetic including structure), and the so‐called 5′‐RNA pocket (three fragment structures) lined by domains 1A, 1B, and 2A which is occupied by the 5′‐end of the substrate RNA in the SARS‐CoV‐2 transcription complex [PDB:7CXM] [7, 11]. These two sites show very high sequence conservation and have good druggability assessment scores. NSP13 is also among the most conserved of the non‐structural proteins in the SARS‐CoV‐2 genome. In addition, a larger fragment binding region located at the interface between the Stalk and Zinc domain was included as, like the nucleotide binding site, this domain binds a large number of fragments (12 fragment structures). This site is proposed to move as part of the catalytic cycle and thus may provide a starting point for allosteric inhibitor discovery [7].

Other binding sites were not included because they either had only one fragment binding (9 out of 19), were estimated too small (3 out of 19), or were estimated too solvent exposed (4 out of 19).

The building of the joint pharmacophore queries is currently done in an interactive mode using the LigandScout 4.5 software. Future work will focus on automating this process further. In essence, the algorithm takes as input the 3D aligned PDB files of the fragment‐monomer pairs for each site and automatically assigns pharmacophore features based on the LigandScout implemented feature detection method [6]. The obtained pharmacophore queries, including the protein excluded volume features, are then merged and interpolated to eliminate redundant features. The result of this step is a joint pharmacophore query for each binding site. The obtained query needs further inspection given that some protein excluded volume features might limit the steric access to pharmacophore features. At this stage, it is unclear how the protein excluded volume constraint should be best handled. Protein residues based on one fragment‐protein complex might have a different conformation in another fragment‐protein complex in order for the accessible free spaces to be mutually exclusive. It is thus unclear how to best specify the excluded volume. This challenge might limit the application cases for which the method will be applicable. It is recommended to remove such protein excluded volume features using the editing functions in LigandScout. It is also noteworthy to state that herein joint pharmacophores were built including solvent molecules. For comparison, it might be of interest in the future to also build queries excluding the solvent molecules.

The last step is the virtual screening itself using the LigandScout XT virtual screening software that enables screening of ultra‐large compound databases of up to 10 billion compounds. The fragment‐screening option was used, which allows selecting hits satisfying a minimum number of query features. In addition, the best conformer hit option was used to allow identification of the best matching conformer.

### 3.2 Virtual Screening

In this study, we virtually screened the J&J corporate screening collection of ~1.5 million in‐house physically available compounds. FragmentScout searches were performed for all three pockets. To provide a comparison for performance, Glide docking was run for the nucleotide and 5′‐RNA binding pockets. Glide docking was not performed for the Stalk‐Zinc domain interface site, as it appears structurally less favorable. Table 1 gives an overview of key parameters for the virtual screens. Given that the J&J corporate screening collection is a drug‐lead‐like screening collection, no triaging of the virtual screening hits was performed. Also, no chemical structure clustering was performed to eliminate possible near‐similar hits. The virtual screening hit lists were respectively sorted according to the pharmacophore‐fit score, and up to ~1000 top‐scoring virtual hits from FragmentScout were kept per site for experimental testing, yielding a final number of 4348 available unique virtual hit compounds, including 1864 hits from Glide docking (see Methods).

**TABLE 1:** Key parameters of the LigandScout XT and Glide SP virtual screens.

Method	Binding site	Initial number of in silico hits	Selected number of in silico hits	Scores for selected in silico hits	Comment
FragmentScout	Nucleotide	3384	1000	96.68–76.30	22 features; 7 minimum required
FragmentScout	5′ RNA	10,969	1000	125.86–105.61	12 features; 9 minimum required
FragmentScout	Stalk‐Zinc	763	763	85.73–74.81	15 features; 7 minimum required
Glide SP	Nucleotide	8145	589	−10.73/−8 kcal/mol	5 constraints; 1 minimum required
Glide SP	5′ RNA	26,111	1275	−9.88/−8 kcal/mol	3 constraints; 1 minimum required

### 3.3 Virtual Screen Hit Confirmation

**FIGURE 2:**
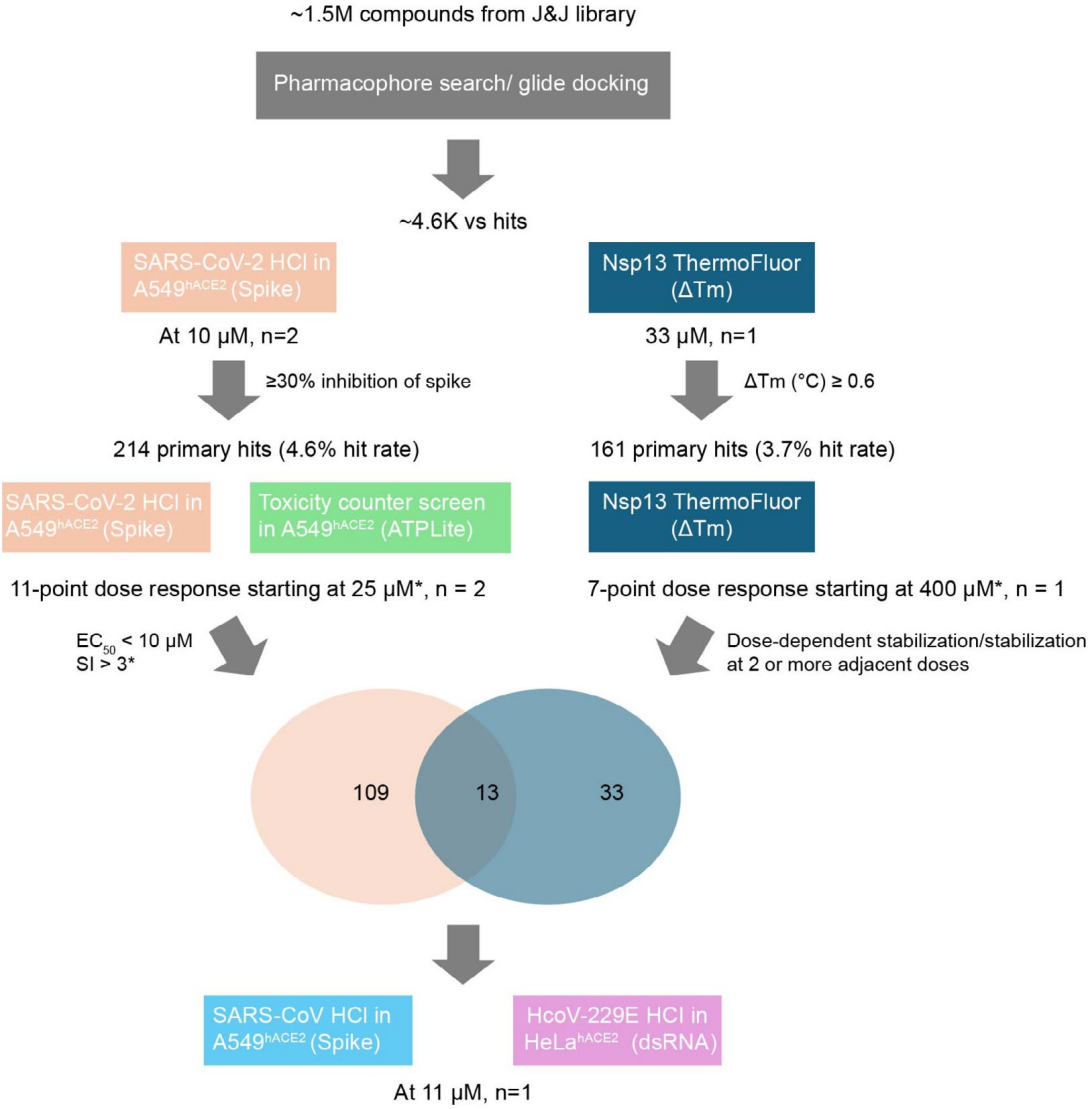
High level flow of the in vitro Nsp13 hit finding cascade. After selecting unique virtual NSP13 hits through the VS workflows, available compounds were confirmed by single‐dose testing at 10 μM (MOI of 0.175) against SARS‐CoV‐2 in a HCI assay in A549‐hACE2 cells (spike stain) and at 33 μM in an NSP13 ThermoFluor assay. Active compounds in the cellular assay (> 30% inhibition of spike) and in the ThermoFluor assay (Δ*T*_m_ ≥ 0.6°C) were selected for dose response testing in the same assays. At this stage, a toxicity counter screen (ATPlite toxicity) was performed in parallel to the cellular antiviral assay. Active compounds in the cellular dose response assay (EC_50_ < 10 μM with either SI > 3 or viability > 70% at highest tested concentration) and in the dose response ThermoFluor assay (clear dose‐dependent stabilization or stabilization with Δ*T*_m_ ≥ 0.6°C for two or more adjacent points) were selected. Compounds selected in both the dose response cellular and biophysical assay were considered as SARS‐CoV‐2 validated hits through the cascade. These hits were then finally tested against other coronaviruses as well (HCoV‐229E and SARS‐CoV) in cell‐based assays. *Highest test concentration used unless not enough compound was available, then a lower start concentration was chosen. EC_50_, 50% effective concentration; HCI, high‐content imaging; *n*, number of replicates in each step; SI, selectivity index; Δ*T*_m_ melting temperature changes.

Of the approximately 4348 virtual hits identified through the virtual screening process, the available candidates were further investigated in vitro using cellular antiviral and biophysical ThermoFluor assays, as outlined in the hit‐finding cascade illustrated in Figure 2.

**FIGURE 3:**
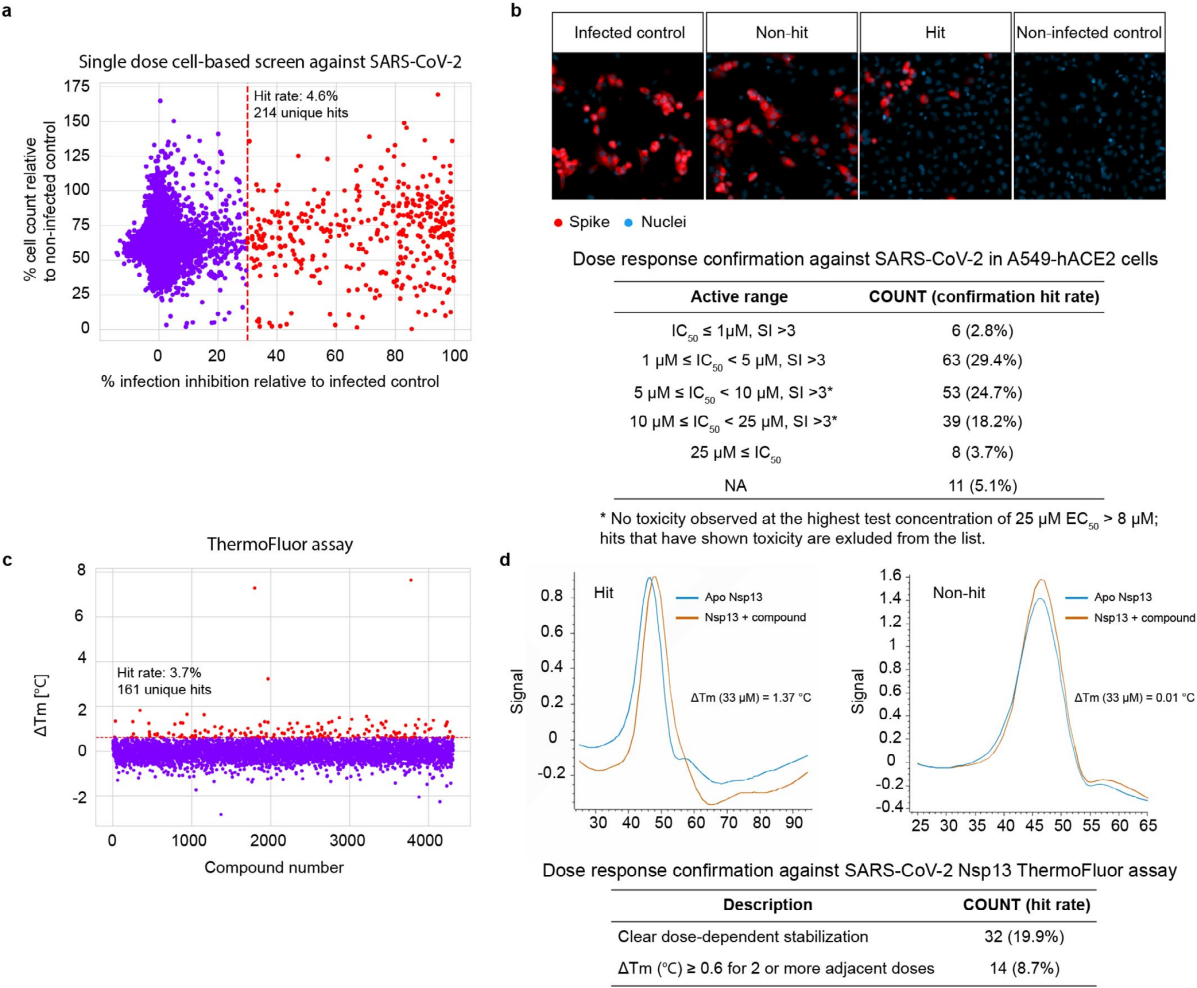
Confirmation of hits in both cellular and biophysical Nsp13 ThermoFluor assays. (a) Hit confirmation against SARS‐CoV‐2 was performed in the A549‐hACE2 cellular assay at single dose using high‐content imaging (HCI) as the readout; The red dashed line indicates the threshold for hit selection and corresponds to inhibition of cells positive for spike of 30% or more. Representative HCI results are shown in (b). These results demonstrate viral infection through spike‐specific antibody staining (red), with nuclei stained by Hoechst (blue). Primary hits selected during the single dose screen were retested in dose response to determine the potency against SARS‐CoV‐2. Potency was calculated based on the percentage of inhibition of cells positive for spike staining. The range of cellular activity for the hits identified from the primary confirmation is listed in the table below the figure. (c) Hit confirmation in the Nsp13 ThermoFluor biophysical assay was conducted with the thermal shift stabilized by the compound serving as the readout. The red dashed line indicates the threshold for hit selection and corresponds to a Δ*T*_m_ of 0.6°C or more. Representative results indicating the melting temperature shifts for hit and non‐hit compounds are shown in (d) and the dose response confirmation of hit rates is provided in the table below.

The cell‐based HCI assay, as previously described [35] was used to assess the antiviral activity of the compounds directly against SARS‐CoV‐2, at a single dose of 10 μM in duplicate (see Figure 3a). Compounds demonstrating over 30% inhibition in the HCI single‐dose assay were selected for further dose–response confirmation and parallel cell toxicity assessment. Initial single‐dose confirmation yielded a hit rate of 4.6%, thereby identifying 214 unique primary hits. Subsequent dose–response confirmation of these hits identified six compounds with an EC_50_ below 1 μM (2.8% hit rate), 63 compounds showing activity between 1 and 5 μM (29.4% hit rate), and 53 compounds with activity ranging from 5 to 10 μM (24.7% hit rate). An overview is shown in Figure 3b. All above‐mentioned hits have shown either more ≥ 70% viability at the highest concentration tested (for compounds with EC_50_ ≥ 8.3 μM) or have a selectivity index ≥ 3 (Figure 3).

The available VS hits were also screened in parallel in a biophysical Nsp13 ThermoFluor assay, initially at a single dose of 33 μM, which yielded a hit rate of 3.7%. Hits were re‐tested in dose response, and the compounds that induced either clear dose‐dependent stabilization or stabilization at 3 or more adjacent doses were selected for follow‐up (see Figure 3 and Figure S1).

In summary, the cell‐based antiviral assay identified 122 unique hits with potency IC_50_ values ≤ 10 μM with either no toxicity or acceptable toxicity (EC_50_ < 10 μM with either SI > 3 or viability > 70% at highest tested concentration). In the biophysical assay, significant thermal shift results were observed for 46 unique compounds. Combining both assays, 13 compounds (Figure S2) were selected as validated non‐toxic hits with clear antiviral activity and clear stabilization of NSP13 protein via ThermoFluor (see above for specific criteria).

**FIGURE 4:**
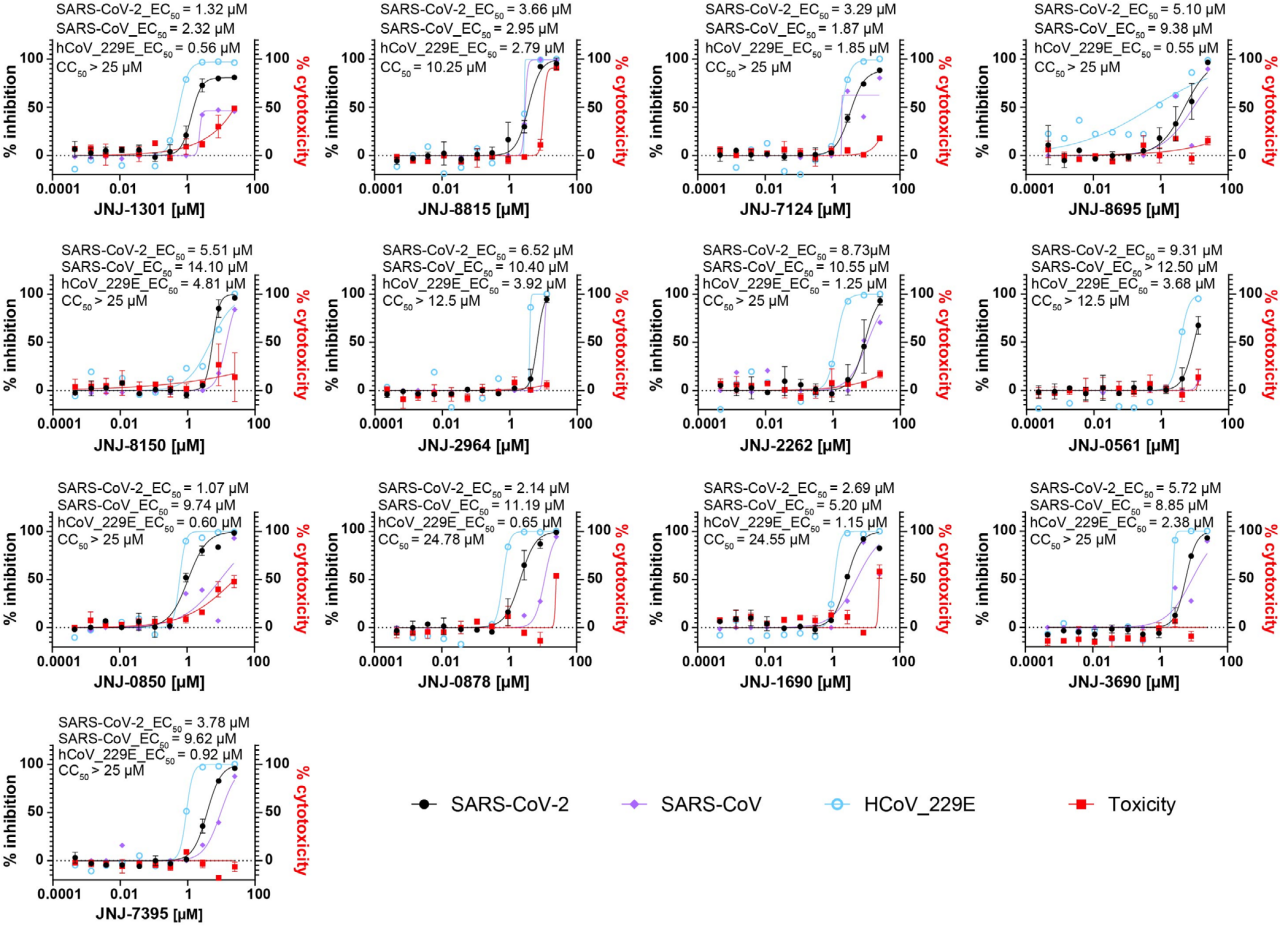
Antiviral activity against SARS‐CoV‐2 (black full circles; spike stain), SARS‐CoV (purple diamond: spike stain), HCoV‐229E (cyan empty circles, dsRNA stain) and corresponding cell toxicity (red full squares, ATPLite) dose response curves in A549‐hACE2 ThermoFluor of the 13 selected hits. Error bars (standard deviation) are shown when *n* = 2 (SARS‐CoV‐2 and toxicity).

The antiviral activity of 13 compounds against SARS‐CoV and the common human coronavirus HCoV‐229E was assessed using cell‐based HCI assays. As indicated in Table 3 and Figure 4, all compounds except for JNJ‐0561, which exhibited no activity against SARS‐CoV, demonstrated antiviral efficacy against both SARS‐CoV and HCoV‐229E, with some variability in potency across the three viruses. Generally, most compounds showed equal or higher potency against HCoV‐229E compared to SARS‐CoV‐2, while the trend was reversed for SARS‐CoV. The high conservation of the screened Nsp13 pockets makes it plausible that compounds tend to be equipotent against different HCoVs.

The analysis of the origin of the hits according to the virtual screening method and the binding site is shown in Table 2. The analysis reveals that one compound appears in the FragmentScout virtual screen to hit both the Stalk‐Zinc and 5′‐RNA sites. Overall, the FragmentScout virtual screen approach performs better than the Glide SP virtual screen, both for the 5′‐RNA and the nucleotide pockets. Moreover, with both methods, more hits were found for the 5′‐RNA pocket compared to the nucleotide pocket. This finding is in line with the better reported druggability score for the former pocket [7]. The large number of hits identified by the FragmentScout virtual screen for the Stalk‐Zinc site corresponds to the presence of larger chemical structure clusters in this set; six compounds of one of the clusters are in the final validated hits.

**TABLE 2:** Origin of hits according to virtual screening method and binding site.

Method	Binding site	#Hits cell‐based assay	#Hits ThermoFluor assay	#Final validated hits
FragmentScout	Nucleotide	21	5	1
FragmentScout	5′ RNA	26	13	5
FragmentScout	Stalk‐Zinc	69	26	7
Glide SP	Nucleotide	2	0	0
Glide SP	5′ RNA	5	3	1

**TABLE 3:** Broad‐spectrum activity of the 13 hits against SARS‐CoV‐2, SARS‐CoV, and HCoV‐229E.

Compound ID	SARS‐CoV‐2 EC_50_ (μM), *N* = 2	SARS‐CoV EC_50_ (μM), *N* = 1	hCoV‐229E EC_50_ (μM), *N* = 1	Cell toxicity CC_50_ (μM), *N* = 2	Target sites	Method
JNJ‐8815	3.66	2.95	2.79	10.25	Nucleotide	FragmentScout
JNJ‐2262	8.73	10.55	1.25	> 25.00	5′‐RNA	FragmentScout
JNJ‐0850	1.07	9.74	0.60	> 25.00	5′‐RNA	FragmentScout
JNJ‐2964	6.52	10.40	3.92	> 12.50	5′‐RNA	FragmentScout
JNJ‐0561	9.31	> 12.50	3.68	> 12.50	5′‐RNA	FragmentScout
JNJ‐8695	5.10	9.38	0.55	> 25.00	Stalk‐Zinc/5′‐RNA	FragmentScout
JNJ‐7124	3.29	1.87	1.85	> 25.00	Stalk‐Zinc	FragmentScout
JNJ‐3690	5.72	8.85	2.38	> 25.00	Stalk‐Zinc	FragmentScout
JNJ‐8150	5.51	14.10	4.81	> 25.00	Stalk‐Zinc	FragmentScout
JNJ‐0878	2.14	11.19	0.65	24.78	Stalk‐Zinc	FragmentScout
JNJ‐7395	3.79	9.62	0.92	> 25.00	Stalk‐Zinc	FragmentScout
JNJ‐1690	2.69	5.20	1.15	24.55	Stalk‐Zinc	FragmentScout
JNJ‐1301	1.32	2.32	0.56	> 25.00	5′‐RNA	Glide

Abbreviations: CC_50_, 50% cytotoxic concentration; EC_50_, 50% effective concentration; *N*, number of replicates.

## 4 Conclusion

We introduced the new FragmentScout virtual screening workflow using the LigandScout software. FragmentScout makes systematic usage of XChem high‐throughput crystallographic screening of fragment data and enables further data mining of the increasing number of proprietary and public XChem datasets [1]. A promising future direction, as recently highlighted by others [36, 37], exists in extending the approach to computationally modeled fragment poses, including QM/MM computations. Preliminary docking of the fragments of the 5′‐RNA site with a semi‐empirical QM (SQM) method indicates that the resulting pharmacophores are very sensitive to the docked poses and protein structure preparation.

Follow‐up studies on three different additional targets have shown that the definitions of the excluded volumes are a critical factor on the performance of retrospective retrieval experiments. While in the reported study we did not give advanced considerations to the excluded volumes and we only eliminated excluded volume features in the joint pharmacophore queries which blocked the steric access to other types of pharmacophore features, we recommend based on our additional new findings to only use consistent protein conformers for each build of a joint pharmacophore model. Fragments resulting into different conformational states of the target should be grouped per conformational state.

After completion of the study, we also implemented an automated joint pharmacophore building script in LigandScoutXT. The performance of the so‐built joint pharmacophores is identical to the interactive joint pharmacophore queries, and the previously identified hits are also retrieved. The inspection step to eliminate blocking excluded volume features remains interactive. While an implementation exists within the Fragalysis software [4] to automate the supervised clustering of the structures from the high‐throughput crystallization experiments, we expect that this step will remain mainly interactive to decide on the possible distinct conformational states of the targets and to control the merging of spatially adjacent clusters.

Applying FragmentScout pharmacophore and Glide SP docking virtual screening to the SARS‐CoV‐2 NSP13 helicase, we reported the discovery of 13 novel micromolar potent non‐cell toxic inhibitors validated in cellular antiviral assays and a biophysical NSP13 ThermoFluor assay. As the compounds were in silico discovered using the isolated structure of the NSP13 helicase and not using the entire replication complex structure of which NSP13 is part it might be worth validating the 122 cell‐based assay hits further in more complex biophysical assays and structure determination experiments as this may reveal additional starting points. In this study, FragmentScout performed better than the well‐established Glide SP virtual screening for both the NSP13 nucleotide and 5′‐RNA pockets. This emphasizes the value of the information rich XChem datasets.

We also compared the results from this work for the 5′‐RNA pocket with results that were recently published within the critical assessment of computational hit finding experiments (CACHE) challenge #2 for the full RNA pocket [20]. In the CACHE#2 competition, 23 participating teams used protein structure and fragment‐screening data paired with advanced computational and machine learning protocols to each predict up to 100 inhibitory ligands [38]. Across all teams, 1957 compounds were predicted and were subsequently procured from commercial catalogues for testing in biophysical assays. Of these compounds, 0.7% was confirmed to bind to Nsp13 in SPR (surface plasmon resonance) experiments. Five of the 23 computational workflows used a pharmacophore searching step in combination with a high‐throughput docking step to select compounds.

The pharmacophore queries were either generated by manual construction, by analysis of the RNA binding site, or by molecular dynamics analysis of the water molecules in the RNA binding site. The six best performing workflows used fragment growing, active learning, or conventional virtual screening with and without complementary deep‐learning scoring functions. Through follow‐up functional assays, two compound scaffolds were identified that bound NSP13 with a Kd below 10 μM and inhibited in vitro helicase activity.

These two compound scaffolds are different from the 6 scaffolds identified by us for the 5′‐RNA pocket. Altogether, these compounds are now available as hit‐to‐lead compounds for further development of novel antiviral agents. To further compare the two studies and more specifically the hit rates is challenging. The CACHE Challenge#2 experiment focuses on the NSP13 RNA binding site while our study includes two additional binding sites. Also, the interrogated chemical spaces are different. The CACHE Challenge#2 virtually screened the Enamine library while we virtually screened the J&J corporate screening library, which are not directly comparable. The J&J corporate library includes, for instance, a number of compounds from internal antiviral research programs. We observe no chemical similarity among the identified validated hits. Given that the pharmacophore models included in some of CACHE Challenge#2 workflows are not accessible, a comparison of the underlying pharmacophore models is not directly possible. A fundamental difference between the two studies is that some of the CACHE Challenge#2 workflows are multi‐step protocols, integrating up to eight different computational techniques, while the FragmentScout workflow only consists of the clustering, query building, and database searching steps. It will be interesting to evaluate the possibility to combine the FragmentScout output with elements of the more successful CACHE Challenge#2 workflows.

## Author Contributions

J.D., J.X., S.M., and E.J. conceived the project idea. J.D., J.X., T.B., M.K.M., P.A., N.V.d.B., D.P., and A.P. designed and performed wet lab experiments, analyzed, and interpreted the data. S.M., C.P., G.I., T.L., C.‐A.M., S.v.R., and E.J. designed in silico experiments, performed in silico experiments, analyzed, and interpreted the data. J.H., R.F., V.P., H.v.V., E.V.D., and M.v.L. provided intellectual contributions to the overall design of the project. J.D., J.X., S.M., T.B., T.L., C.‐A.M., and E.J. wrote the manuscript. All authors read and approved the manuscript.

## Conflicts of Interest

E.V.D. and M.v.L. have been named inventors in a pending patent application claiming inhibitors of coronavirus (WO 2024008909), which was filed by the applicant Janssen Pharmaceutica NV. J.D., J.X., S.M., T.B., M.K.M., P.A., S.v.R., R.F., V.P., D.P., A.P., E.V.D., H.V.V., M.v.L., and E.J. were/are employees of Johnson & Johnson and may possess stocks of Johnson & Johnson. N.V.d.B. is an employee of Charles River Laboratories, a contract research organization, and may possess stocks of Johnson & Johnson. All other authors declare no competing interests.

## Supporting information


**Data S1:** Supplementary Information.

## Data Availability

The data that support the findings of this study are available from the corresponding author upon reasonable request.
