# Supplementary material for: A New Fragment‐Based Pharmacophore Virtual Screening Workflow Identifies Potent Inhibitors of SARS‐CoV‐2 NSP13 Helicase
Source: J Comput Chem. 2025 Sep 5;46(23):e70201. doi: 10.1002/jcc.70201 (PMC12412281; doi:10.1002/jcc.70201)
Supplement: Supplementary file 1 — Data S1: Supplementary Information. [file JCC-46-0-s001.pdf]

Supplementary Figures

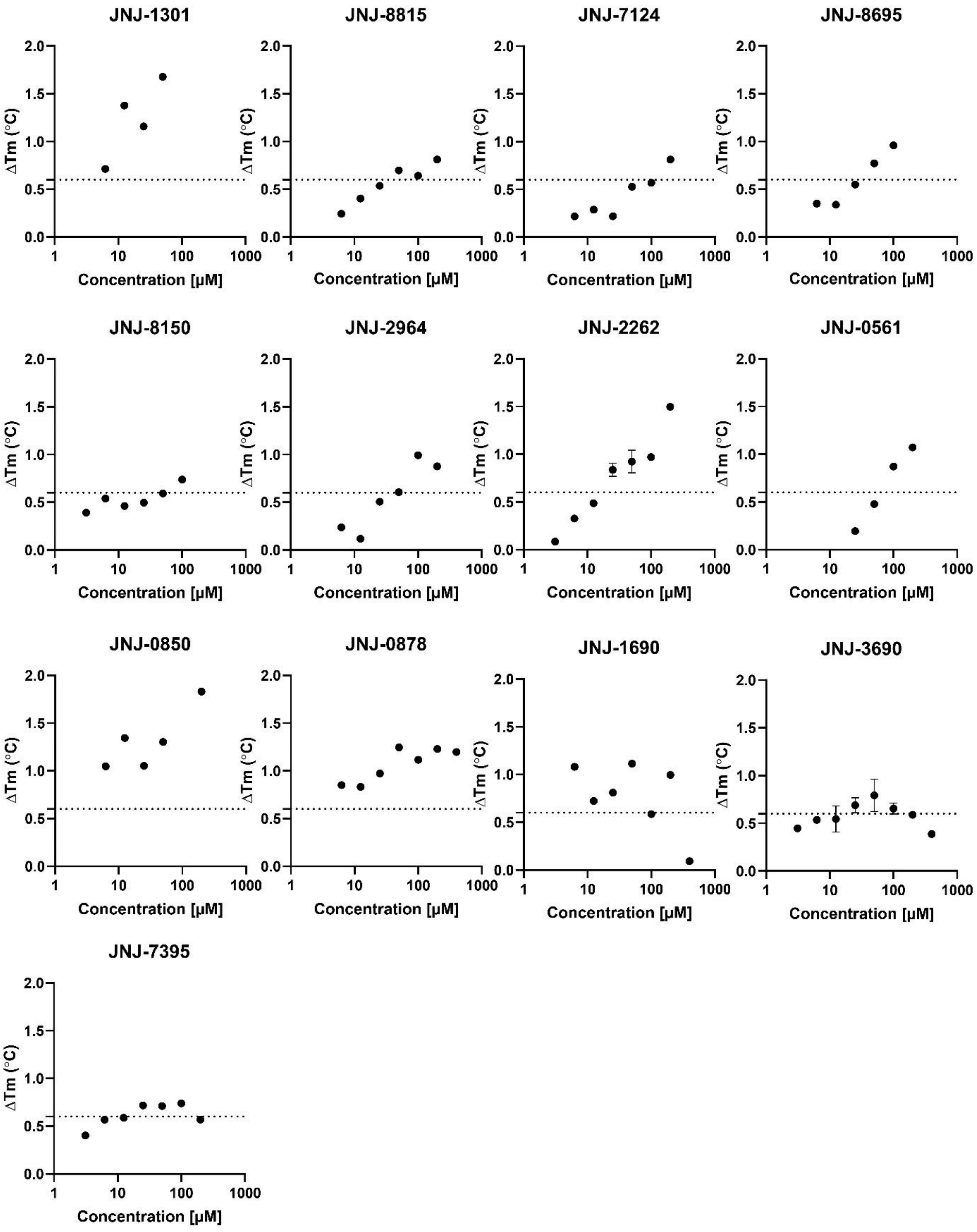

Supplementary Figure 1. ThermoFluor assay ΔTm in function of dose for the 13 selected hits

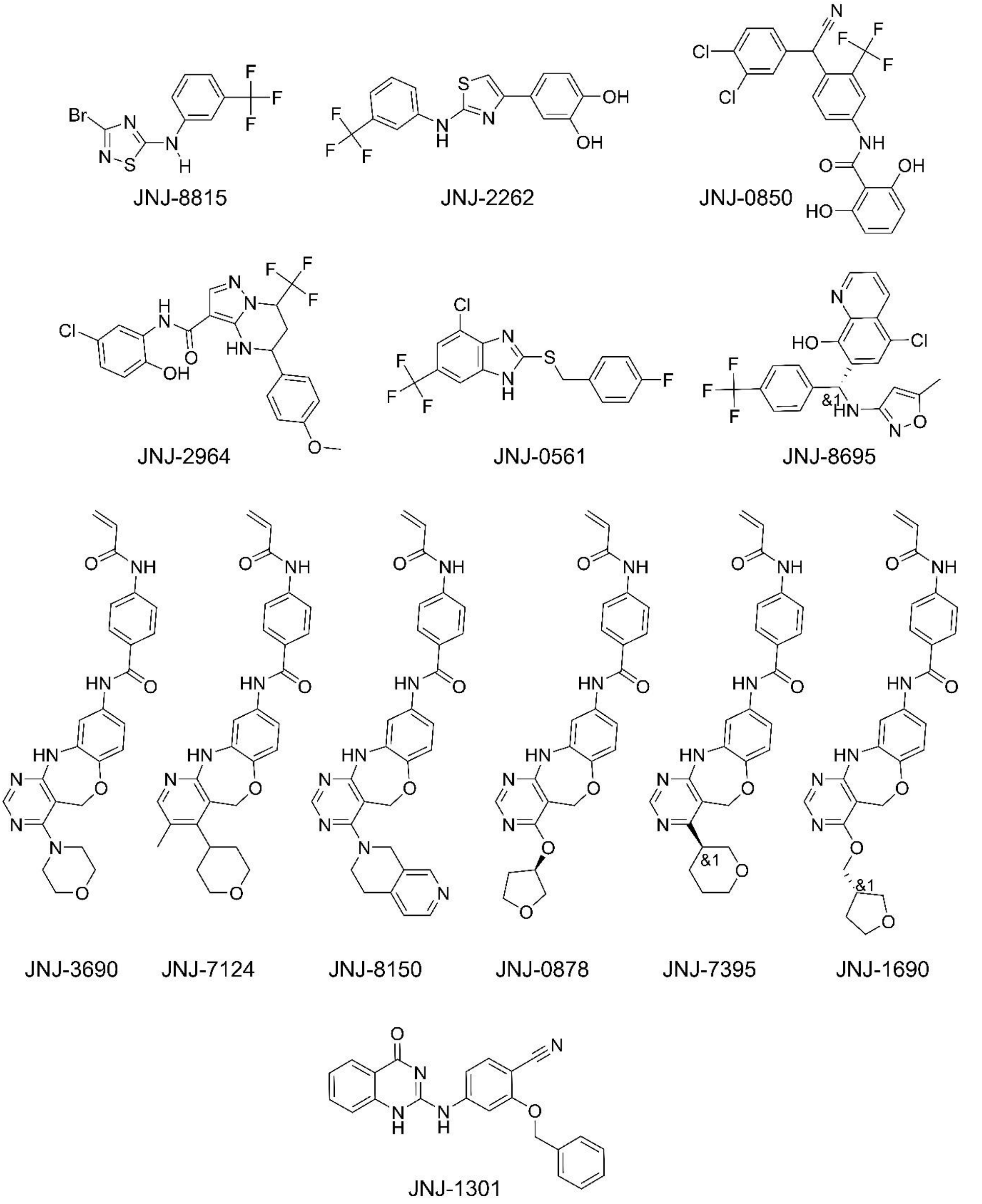

Supplementary Figure 2. Compound ID and structure of the 13 hits against SARS-CoV2, SARS-CoV, HcoV-229E.
